# Development of a Transgenic *Plasmodium berghei* Line (Pb*^pfpkg^*) Expressing the *P. falciparum* cGMP-Dependent Protein Kinase, a Novel Antimalarial Drug Target

**DOI:** 10.1371/journal.pone.0096923

**Published:** 2014-05-07

**Authors:** Rita Tewari, Eva-Maria Patzewitz, Benoit Poulin, Lindsay Stewart, David A. Baker

**Affiliations:** 1 Centre for Genetics and Genomics, School of Life Sciences, Queens Medical Centre, University of Nottingham, Nottingham, United Kingdom; 2 Department of Pathogen Molecular Biology, Faculty of Infectious and Tropical Diseases, London School of Hygiene & Tropical Medicine, London, United Kingdom; Liverpool School of Tropical Medicine, United Kingdom

## Abstract

With the inevitable selection of resistance to antimalarial drugs in treated populations, there is a need for new medicines to enter the clinic and new targets to progress through the drug discovery pipeline. In this study we set out to develop a transgenic rodent model for testing inhibitors of the *Plasmodium falciparum* cyclic GMP-dependent kinase *in vivo*. A model was needed that would allow us to investigate whether differences in amino acid sequence of this enzyme between species influences *in vivo* efficacy. Here we report the successful development of a transgenic *P. berghei* line in which the cyclic GMP-dependent protein kinase (PKG) was replaced by the *P. falciparum* orthologue. We demonstrate that the *P. falciparum* orthologue was able to functionally complement the endogenous *P. berghei pkg* gene throughout blood stage development and early sexual development. However, subsequent development in the mosquito was severely compromised. We show that this is due to a defect in the female lineage of the transgenic by using genetic crosses with both male and female deficient *P. berghei* lines. This defect could be due to expression of a female-specific target in the mosquito stages of *P. berghei* that cannot be phosphorylated by the *P. falciparum* kinase. Using a previously reported anti-coccidial inhibitor of the cyclic GMP-dependent protein kinase, we show no difference in *in vivo* efficacy between the transgenic and control *P. berghei* lines. This *in vivo* model will be useful for screening future generations of cyclic GMP-dependent protein kinase inhibitors and allowing us to overcome any species-specific differences in the enzyme primary sequence that would influence *in vivo* efficacy in the rodent model. The approach will also be applicable to *in vivo* testing of other antimalarial compounds where the target is known.

## Introduction

In 2012, malaria caused an estimated 627,000 deaths (with an uncertainty range of 473,000 to 789,000), mostly among African children (http://www.who.int). The malaria burden has fallen dramatically in some countries in recent years, likely due to scaling up of interventions such as vector control programmes and the use of artemisinin combination therapy (ACT) as the first line of treatment. However, it has been firmly established in parts of Southeast Asia that ACT has developed a reduced efficacy in many patients [1], [2] likely heralding resistance to this drug which would be a public health disaster in the absence of alternative treatments. It is therefore imperative that the drug discovery pipeline receives new candidates and delivers products to the clinic.

One potential candidate that has received attention in recent years is the *Plasmodium falciparum* cGMP-dependent protein kinase (PfPKG). PKG has diverse roles across eukaryotes. In mammals PKG is encoded by two distinct genes: *prkg1* (encoding two isoforms, PKGIα and PKGIβ) and *prkg2*, both of which are downstream effectors of nitric oxide-stimulated cGMP signalling and also atrial natriuretic peptide-stimulated cGMP signalling. The PKGI isoforms have major roles in smooth muscle relaxation and platelet aggregation, whereas PKGII is involved in processes such as intestinal secretion, circadian rhythm and endochondreal bone growth [3]. All PKG enzymes comprise a C-terminal catalytic domain and an N-terminal regulatory domain in a single polypeptide. Mammalian PKGs have two cGMP-binding domains in the regulatory domain, whereas the malarial PKG has three functional cGMP-binding domains as well as a degenerate fourth domain which is required for optimal enzyme activation [4], [5]. Binding of cGMP, dramatically changes the conformation of the enzyme [6] allowing substrate binding and transfer of the γ-phosphate of ATP to a serine/threonine residue of the bound substrate.

The PKG enzyme from *Eimeria tenella* was the focus of an earlier anti-coccidial drug discovery program [7]. Highly specific, selective lead compounds were shown to also inhibit *Plasmodium* PKG [8], [9]. These compounds have proven to be excellent tools for investigating the biological role of PKG and cGMP signalling in malaria parasites especially when used in conjunction with transgenic parasites expressing an inhibitor-resistant PKG. The selectivity of these classes of PKG inhibitors (a pyrrole, compound 1 and an imidazopyridine, compound 2) relies on a rare structural feature of the apicomplexan PKG enzyme. It has a threonine residue (with a relatively small side chain) occupying the so-called ‘gatekeeper’ position. The presence of threonine in this position allows inhibitor access to a small hydrophobic pocket adjacent to the ATP-binding site of the kinase [10], [11]. The gatekeeper position of most protein kinases of the AGC superfamily in mammals is occupied by an amino acid with a relatively bulky amino acid (e.g. methionine in human PKG isoforms) which prevents access of the inhibitor to the hydrophobic pocket. These properties have been exploited in a chemical genetic approach to functional analysis of PKG in coccidian and malaria parasites. Recombinant parasite PKGs in which the gatekeeper residue is mutated from threonine to methionine or glutamine are dramatically less sensitive to the inhibitors with IC_50_ values 3–4 logs higher using *in vitro* kinase assays [9], [10]. Transgenic parasites expressing these mutant PKGs are inhibitor resistant. Testing of wild type and transgenic gatekeeper mutant lines in parallel with PKG inhibitors provides a means of providing direct evidence of a role for PKG in a cellular process or differentiation stage of interest. Using this approach we have previously demonstrated a role for PfPKG in sexual development. PKG inhibitors block the initial step of gametogenesis in wild type *P. falciparum* parasites, whereas this process occurs normally in the inhibitor-treated gatekeeper mutant parasites [9]. We have also established a role for PfPKG in asexual blood stage schizont rupture and merozoite egress [12]. Additional work has shown that at least part of the underlying mechanism of PKG inhibition at this life cycle stage is a complete block in the function of the protease PfSUB1 which is essential for merozoite egress [13]. The proteolytic processing of the MSP1 complex and SERA proteins by PfSUB1 is blocked by PKG inhibitors. PKG inhibitors have no effect on the catalytic activity of PfSUB1 or its trafficking to the exonemes; a discrete set of apical organelles from which PfSUB1 is released immediately prior to merozoite egress [14]. Recent work has shown that PKG inhibitors in fact block the release of PfSUB1 from the exonemes as well as blocking discharge of AMA1 from the micronemes onto the merozoite surface; a process which is essential for merozoite invasion of erythrocytes [15].

This first generation of PKG inhibitors showed significant effects on progression of both the asexual blood stage (that causes pathology) and the sexual stage (that mediates transmission) of the malaria parasite life cycle. It has also been reported that PKG has an essential role in the late liver stage of the rodent malaria parasite *P. berghei* [16] and so this kinase has attracted interest in terms of its potential as a drug target. An important step in the early drug discovery pipeline is to test lead compounds for *in vivo* efficacy in the rodent malaria model *P. berghei*. A question which arose as part of these experiments was whether test inhibitors would have equivalent inhibitory effects on the *P. berghei* and *P. falciparum* PKG enzymes. It has previously been reported that interspecies differences between the amino acid sequences of *P. falciparum* falcipains and their *P. berghei* orthologues confer differential inhibitor sensitivity [17], [18].

To address this issue, in the present work we report development of a GFP-expressing transgenic *P. berghei* line in which the rodent malaria PKG was replaced by the *P. falciparum* PKG. Although PfPKG complemented PbPKG throughout the asexual blood stage and the early phases of sexual development, we show that transgenic parasites exhibited markedly reduced ookinete production that was due to a defect in the female-specific lineage. Moreover, while the two lead compounds tested showed no significantly different inhibitory effects of control or the Pb*^pfpkg^* transgenic lines, the newly established line provides an *in vivo* model for testing the next generation of PKG inhibitors and will be valuable for testing inhibitors where the sensitivity of PbPKG and PfPKG recombinant proteins differ.

## Results

### Generation of Pb*^pfpkg^*, a *P. berghei* Transgenic Line in Which PbPKG is Replaced with PfPKG

**Figure 1:**
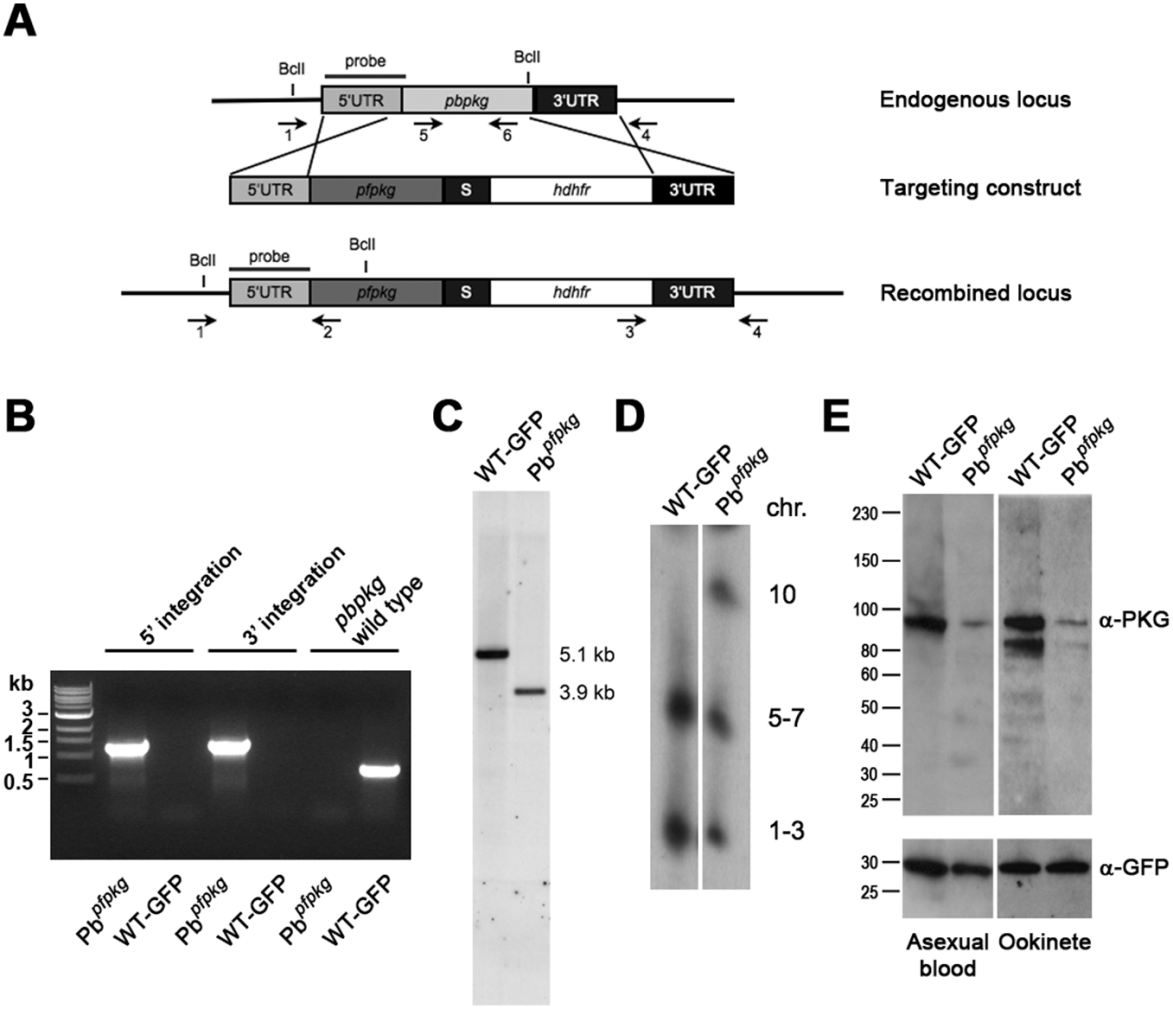
Generation of Pb*^pfpkg^* parasites. A) Schematic diagram of the endogenous *pbpkg* locus, the targeting construct and the transgenic *pb^pfpkg^* locus. Areas of 5′UTR and 3′UTR cloned into the targeting vector are indicated, S = spacer. Arrows 1–6 indicate binding sites for primers used in diagnostic PCR. Primers 1 and 2 were used to detect 5′ integration. Primers 3 and 4 were used to determine 3′ integration. Primers 5 and 6 bind specifically to the endogenous *pbpkg* and are used to confirm absence of the endogenous gene in the transgenic line. The area homologous to the probe used in Southern blotting and *Bcl*I restriction sites used for diagnostic digest are indicated. B) Diagnostic PCR used to determine integration of the targeting construct into the Pb*^pfpkg^* transgenic line. C) Southern blot following *Bcl*I digest shows integration of the targeting construct as a specific 3.9 kb band and absence of the endogenous 5.1 kb band in the transgenic line (Pb*^pfpkg^*) in comparison to wild type (WT-GFP). D) PFGE of wild type (WT-GFP) and mutant parasite (Pb*^pfpkg^*) confirms integration into the correct chromosome. E) Western blot of asexual blood and ookinete stages confirm expression of PfPKG in the transgenic line (the transgenic PfPKG bands are 25.8% and 21.8% of the PbPKG band in the WT-GFP line in asexual blood and ookinete stages respectively).

We generated a transgenic rodent *P. berghei* parasite line Pb*^pfpkg^*, in which the endogenous *pbpkg* gene (*PBANKA_100820*) was replaced with that of the human parasite *P. falciparum* (*PF3D7_1436600*) (**Figure 1A**). The *P. berghei* ANKA 507 clone 1 constitutively expressing GFP (WT-GFP) was used for transfection [19]. Following drug selection and cloning of the drug resistant parasite the clones were genotyped; with integration of the targeting vector confirmed by the presence of a 1.2 kb PCR band for each 5′ and 3′ integration event in the transgenic line using integration-specific primer pairs (**Figure 1B**). An additional PCR specific for the endogenous *pbpkg* was performed to confirm the absence of the endogenous gene. As expected, this PCR resulted in the detection of a 0.7 kb band only when wild type gDNA was used (**Figure 1B**). Integration was further confirmed by Southern blotting following a diagnostic digest with *Bcl*I using a probe corresponding to the 5′UTR (**Figure 1C**). The blot confirmed the absence of the diagnostic 5.1 kb band expected for the endogenous *pbpkg* locus and the presence of a 3.9 kb band in the Pb*^pfpkg^* line. Integration into the correct chromosome (chromosome 10) was further confirmed by pulsed-field gel electrophoresis (PFGE) (**Figure 1D**). Western blotting using an anti-human PKG antibody recognising both PbPKG and PfPKG confirmed that PfPKG is expressed in the transgenic parasites in asexual blood and ookinete stages (**Figure 1E** **)**.

### Development of the Transgenic Pb*^pfpkg^* Parasite through the Life Cycle

**Figure 2:**
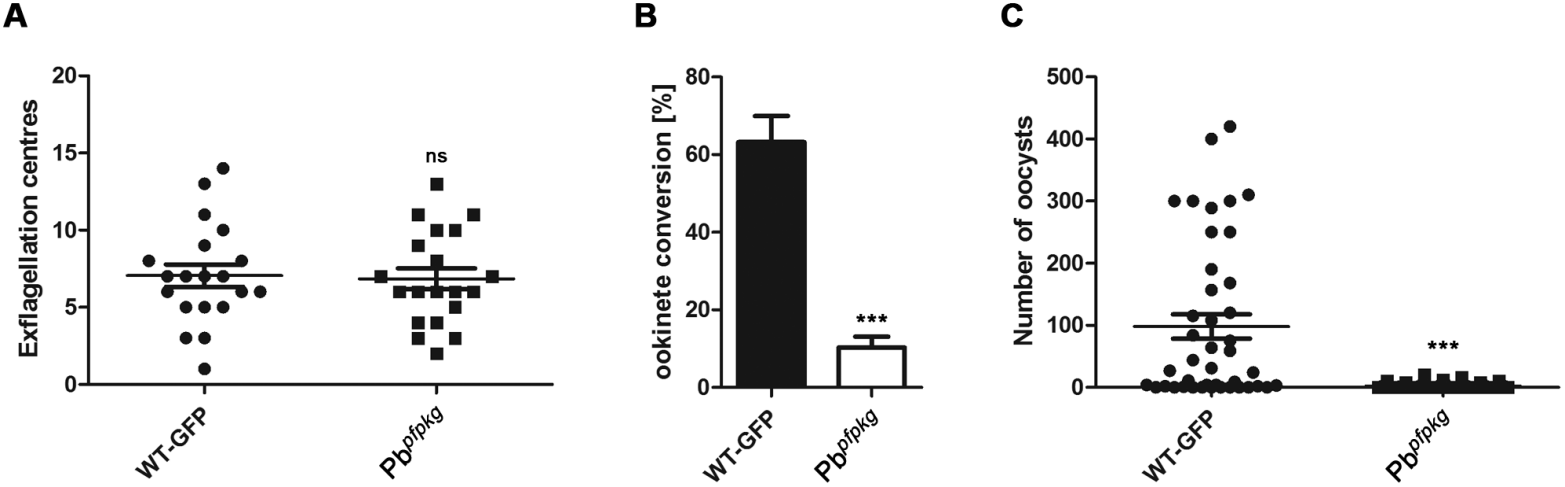
Parasite development in the mosquito. A) Exflagellation in wild type (WT-GFP) and transgenic Pb*^pfpkg^* parasites. The mean number of exflagellation centres was 7.0 for wild type and 6.8 for Pb*^pfpkg^* parasites. Bar, mean ± SEM (Mann-Whitney U test: ns, not significant, p>0.05 compared to WT-GFP). B) Ookinete conversion in wild type (WT-GFP) and transgenic Pb*^pfpkg^* parasites. Wild type conversion was 63% and Pb*^pfpkg^* conversion was 10%. Data shown as mean ± SEM (Mann-Whitney U test: ***, p<0.001 compared to WT-GFP). C) Gut oocyst numbers in wild type (WT-GFP) and transgenic Pb*^pfpkg^* parasites. Wild type infected guts contained 100 oocysts and Pb*^pfpkg^* infected guts with less than 10 oocysts. Bar, mean ± SEM (Mann-Whitney U test: ***, p<0.001 compared to WT-GFP).

Blood stages and gametocytes developed normally as assessed by Giemsa stained blood stage parasites (data not shown). Exflagellation in mutant and control parasites expressing GFP *in vitro* showed no significant difference between the two lines (**Figure 2A**). However, ookinete conversion after 24 hours *in vitro* was markedly decreased in the transgenic line (**Figure 2B**), with a conversion rate of 63% in wild type and only 10% in Pb*^pfpkg^* parasites.

When mosquitoes were allowed to feed on mice infected with either control or mutant parasite lines, the number of oocysts observed on day 14 post infection (p.i.) was also markedly decreased in the Pb*^pfpkg^* line (**Figure 2C**). Next, we tested for the presence of sporozoites in salivary glands on day 21 p.i., but did not observe any sporozoites in salivary glands of mosquitoes infected with the mutant line (data not shown). In three independent bite back experiments 3/3 mice bitten by mosquitoes infected with the control parasite developed a blood stage infection on day 4–5 post bite back. Mice bitten by mosquitoes infected with the mutant were monitored until day 14 post bite back and 0/3 mice became positive. Together these experiments show that while PfPKG can functionally complement PbPKG in asexual blood stages of Pb*^pfpkg^* parasites, as well as in gametocyte development and male gametogenesis, replacement of the endogenous gene with PfPKG leads to a decreased conversion into ookinete stages, reduced oocyst production and no detectable sporozoites causing a block of parasite transmission in the mosquito.

### Genetic Complementation Experiment with Pb*^pfpkg^*

**Figure 3:**
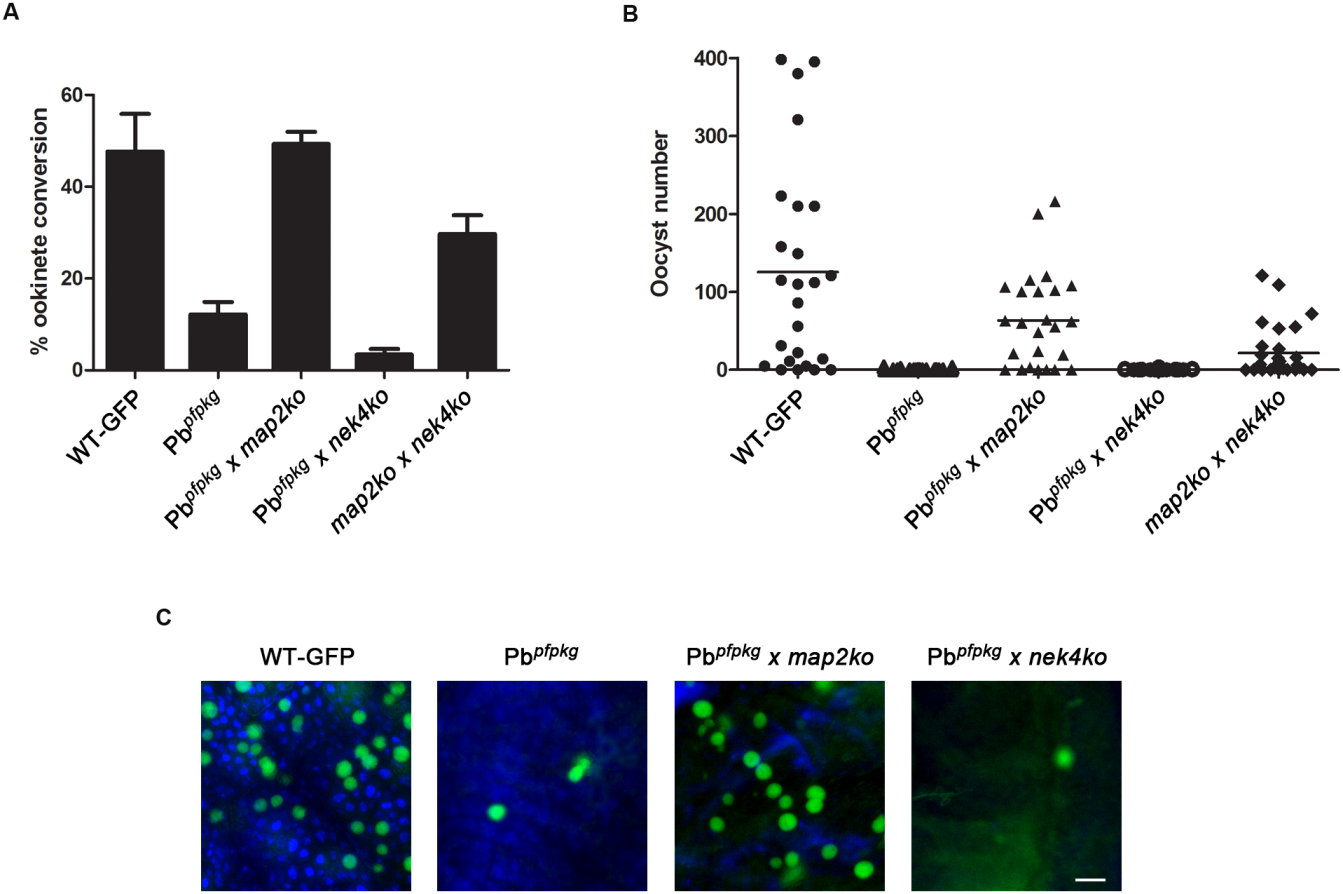
Genetic crossing of Pb*^pfpkg^* parasites in ookinete and oocyst stages. A) Ookinete conversion scored after crossing Pb*^pfpkg^* mutants with either female-defective (*nek4ko*) or male-defective (*map2ko*) mutants. Wild-type parasites (WT-GFP) and *map2ko* mutant crossed with *nek4ko* line were used as controls. Data shown as mean ± SEM. B) For oocyst rescue, mosquitoes were fed on mice infected with WT-GFP or Pb*^pfpkg^* mutant alone or coinfected with Pb*^pfpkg^* and either a *map2ko* mutant or a *nek4ko* mutant. Bar, arithmetic mean. C) Representative images of mosquito gut from crosses in B). Scale bar, 100 µm.

To determine if the decrease in ookinete conversion, oocyst production and subsequent block in sporogony is a result of a sex-specific defect, we performed genetic crosses as described earlier to investigate mutants with ookinete defects [20], [21]. Genetic crossing with lines that are deficient in either male (*map2ko*) or female (*nek4ko*) gametes showed that only the male mutant *map2ko* line was able to rescue both the ookinete conversion (**Figure 3A**) and oocyst numbers (**Figure 3B, C**) whereas the female mutant did not show rescue of the phenotype. These experiments demonstrate that the inability of *Pb^pfpkg^* to complete mosquito stage development is due to a defect in the female lineage.

### Effects of PKG Inhibitors on Transgenic Pb*^pfpkg^* Development

We next tested exflagellation and ookinete conversion of both wild type and transgenic Pb*^pfpkg^* parasites in the presence of two ATP-competitive PKG inhibitors: compound 1 (a trisubstituted pyrrole) and compound 2 (an imidazopyridine). PKG has previously been shown to be the primary target of these inhibitors during the asexual and sexual stages of the malaria parasite life cycle [8], [9], [12], [22], [23].

**Figure 4:**
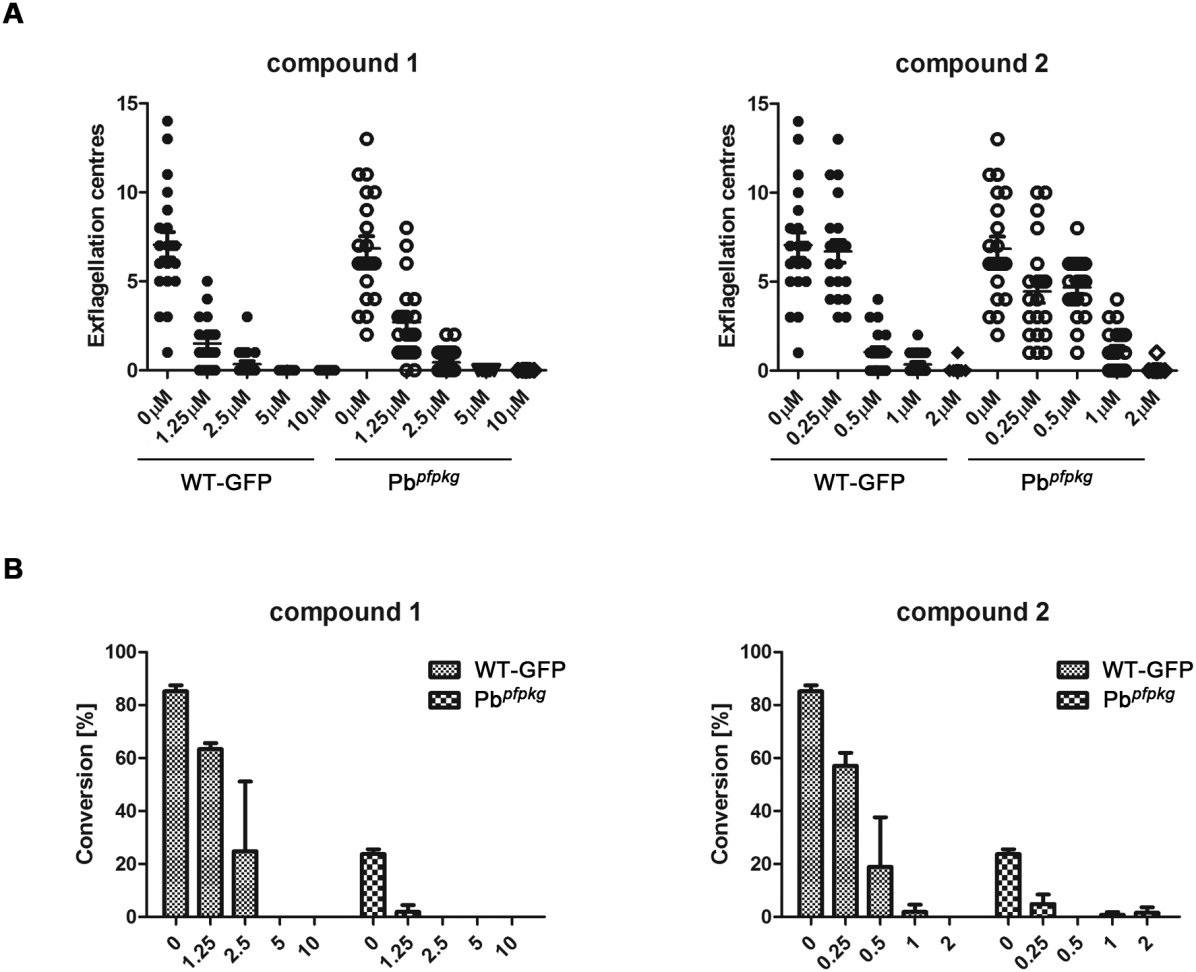
Inhibition of exflagellation and ookinete conversion using PKG inhibitors. A) Inhibition of exflagellation: exflagellation was measured in the presence of increasing concentrations of compound 1 and compound 2 on both WT GFP line expressing PbPKG (WT-GFP) and the transgenic line expressing PfPKG (Pb*^pfpkg^*) Bar, arithmetic mean ±SEM. B) Inhibition of ookinete conversion: ookinete conversion was determined in the presence of increasing concentrations of compound 1 and compound 2 in WT-GFP and the transgenic Pb*^pfpkg^* line as above. Data shown as mean ± SD.

Both compounds had a marked effect on exflagellation in control and mutant Pb*^pfpkg^* transgenic parasites. Compound 2 was more effective against both lines leading to a complete inhibition of exflagellation at a concentration of 2 µM, whereas compound 1 led to a complete inhibition at a concentration of 5 µM (**Figure 4A**).

In addition, ookinete conversion was also inhibited in control and Pb*^pfpkg^* parasites by both compound 1 and compound 2 (**Figure 4B**), with compound 2 again being the more effective inhibitor. While a concentration of 2 µM was again enough to completely abolish ookinete conversion for compound 2, the same effect was seen only at a concentration of 5 µM for compound 1. The IC_50_ values for the inhibition of ookinete conversion by compound 1 were 1.25 µM and 2.5 µM for control and Pb*^pfpkg^* parasites, respectively, and 0.5 µM and 1.0 µM for compound 2. As ookinete conversion was already markedly decreased in Pb*^pfpkg^* parasites compared to wild type, it was not possible to determine whether either inhibitor had a more pronounced effect on the transgenic Pb*^pfpkg^* parasites and whether there was a differential effect on PfPKG and PbPKG.

### *In vivo* Effects of Compound 2 on Control and Transgenic Pb*^pfpkg^ P. berghei* Lines

**Figure 5:**
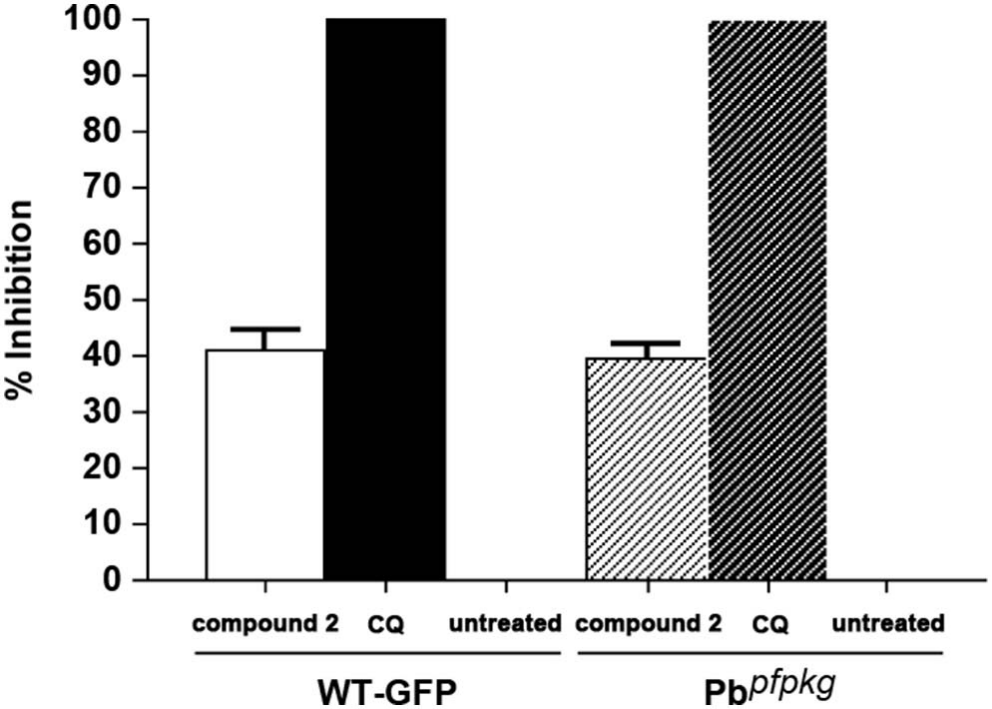
*In vivo* blood stage efficacy test using compound 2. A Peters 4 days test was carried out using both WT-GFP and transgenic Pb*^pfpkg^* with the PKG inhibitor compound 2. Both WT-GFP and Pb*^pfpkg^* transgenic parasites showed a 40% reduction in blood stage parasitaemia with compound 2 given at 25 mg/kg twice daily via the oral route. Chloroquine (CQ) was used as control showed a 100% reduction in blood stage parasitaemia in mice after a single daily oral dose of 10 mg/kg.

We next compared the *in vivo* efficacy of compound 2 in control and transgenic Pb*^pfpkg^* parasites using a standard Peters four day test [24]. This assay was used as a proof of concept for use of the transgenic line as a model for future *in vivo* efficacy testing of PKG inhibitors. It was found that compound 2 gave an equivalent reduction in blood stage parasitaemia of (40–50%) in both lines (**Figure 5**).

## Discussion

The malaria parasite protein kinase PKG has essential roles in multiple life cycle stages both in the mammalian host and insect vector [25]. Several recent studies have demonstrated its regulatory functions in apical organelle release and proteolytic processing events required for merozoite egress [13], [15], [22], gametocyte activation/gamete egress [9] and in ookinete [23] and in liver stage development [16]. This suggests that this kinase may have diverse targets/substrates during these various stages of parasite development and they may be stage specific. These varied functions make PKG a potentially significant antimalarial target. Functional kinome studies have demonstrated that the *pkg* gene is refractory to deletion both in *P. falciparum* and *P. berghei* [26], [27].

The results generated here with the *P. berghei* transgenic line expressing the *P. falciparum* PKG (Pb*^pfpkg^*) demonstrate that PfPKG can complement the function of *P. berghei* PKG during asexual blood stage and early sexual development; stages in which PKG is known to be essential [9], [25], [26], [27]. However, its function is compromised during ookinete development and the defect even more pronounced in terms of oocyst and sporozoite formation. This perhaps suggests that the *P. falciparum* PKG is not able to interact with one or more *P. berghei*-specific downstream targets resulting in inefficient functional complementation at the latter mosquito stages of parasite development. Among the various possible explanations for the transgenic line being unable to complete development in the mosquito, a sex-specific defect was a potential candidate. A genetic complementation rescue experiment demonstrated that the defect was derived from the female lineage, suggesting a female gamete-specific *P. berghei* substrate that cannot be phosphorylated by the *P. falciparum* PKG.

Previous functional studies using two compounds to inhibit parasite development [9], [15], [25] were validated in our proof of principle study and confirmed that this transgenic line is useful to study the effects of *P. falciparum* PKG inhibitors on blood stage and gametocyte development in an *in vivo* context as well as on exflagellation. In addition, *in vivo* testing of PKG inhibitors on the Pb*^pfpkg^* transgenic line showed similar levels of efficacy to those obtained with the control line. The transgenic line will be also be a very useful tool for *in vivo* screening of future generations of PKG inhibitors where the compounds show differential inhibitory effects using *in vitro* PfPKG and PbPKG kinase assays.

## Materials and Methods

### Ethics Statement

All animal work was approved by the United Kingdom Home Office and passed an ethical review process. All work carried out (under project license permit numbers 40/3344 at University of Nottingham and 70/6996 at LSHTM) was in compliance with European Directive 86/609/EEC for the protection of animals used for experimental purposes and in accordance with the United Kingdom “Animals (Scientific Procedures) Act 1986”.

### Animals

Tuck’s Original (TO) (Harlan) outbred mice were used for all experiments except for Peters four day tests where BALB/c (Charles River) were used.

### Generation of Replacement Targeting Construct

The replacement construct for *pbpkg* was constructed using the plasmid pOB116 containing *hdhfr* as selectable marker for transgenic parasites. Upstream of the selectable marker cassette a 979 bp fragment upstream of the *pbpkg* ATG start codon was amplified using the primers *Apa*IPKG 5′-CCCCGGGCCCCCCCAATTGACGCCATTTCAACTGAG-3′ and *Sac*IIPKG 5′-CCCCCCGCGGTTTTCTTATCTTCCAATCTCGTTAAC-3′ and inserted using *Apa*I and *Sac*II restriction sites. The *pfpkg* coding region was amplified from cDNA using the primer pair *Sac*IIPfPKG 5′-CCCCCCGCGGATGGAAGAAGATGATAATCTAAAAAAAGGG-3′ and *Bgl*IIPfPKG 5′-CCCCAGATCTTTAAAAATCTATGTCCCAGTTGTCTTC-3′ and inserted between *Sac*II and *Bgl*II restriction sites. A 438 bp spacer region corresponding to part of the *pbpkg* 3′UTR downstream of the stop codon was cloned between *Bgl*II and *Pst*I restriction sites using the primers *Bgl*IIPKG 5′-CCCCAGATCTTTGAGGAAAGTGATAAAAACAGAG-3′and *Pst*IPKG 5′-CCCCCTGCAGCATATATACATGTGTGCATTTAC-3′. Downstream of the selectable marker cassette a 1100 bp region of the 3′UTR was cloned into *Xho*I and *Not*I restriction sites using primers *Xho*IPKG 5′-CCCCCTCGAGTTGAGGAAAGTGATAAAAACAGAG-3′ and *Not*1PKG5′-CCCCGCGGCCGCCTGTCAGATAAAGAAATTTCGTTAAAAC-3′.

The linear targeting sequence was released using *Apa*I/*Not*I.

For transfection, the *P. berghei* ANKA 507 clone 1 constitutively expressing GFP was used as previously described [19]. The ANKA 507 clone 1 was used as a control line throughout this study. Following electroporation parasites were mixed immediately with 150 µl reticulocyte-rich blood from a naïve, phenylhydrazine-treated mouse and incubated for 30 min at 37°C. Following incubation, the electroporated parasites were injected intraperitoneally (i.p.) into a naïve mouse. From day 1 post infection, transgenic parasites were selected by treating mice for four days with 7 mg/ml pyrimethamine (Sigma) in their drinking water. Mice were monitored for 15 days and drug selection was repeated after passage to a second mouse. Resistant parasites obtained after the second pressure were cloned by limiting dilution cloning and used for genotyping.

### Genotypic Analyses of Transgenic Parasites

To confirm correct integration of the targeting construct, diagnostic PCR was set up using primers INT-PKG-5 5′-CCGTATATACTAGGATCATATATGTTC-3′ and PfPKG-5-INT-R 5′-CAAAGAATTGCATATAATTAGACAGAGTC-3′ for 5′ integration and 539 5′-CAATGATTCATAAATAGTTGGACTTG-3′ and Int-PKG-3′R 5′-GTTAAAACAAATATATTGGCAGGTACTAC-3′ for 3′-integration, both giving a 1.2 kb band in the transgenic parasites. As a control the primers PbPKG01 5′-CATATATGCTGAATATACATGTGTACTTTG-3′ and PbPKG02 5′-GTGCATAAATACGCATAGAAATAGAAG-3′ were used, which amplify a 0.7 kb fragment in of the wild type.

Southern blot was performed following digestion of genomic DNA of wild type and transgenic parasites with *Bcl*I. Digested DNA was separated on a 0.8% agarose gel before being transferred onto a nylon membrane and being probed with a probe homologous to the 5′UTR. The probe was generated using the Amersham AlkPhos direct labelling and detection kit (GE Healthcare) according to manufacturer’s instructions.

Chromosomes of wild type and transgenic parasites were separated by pulsed field gel electrophoresis (PFGE) using a CHEF-DR III system (Bio Rad). A linear ramp of 60–500 s for 72 h at 4 V/cm was used and gels were blotted onto a nylon membrane. A probe recognizing both the resistance cassette in the targeting vector and the 3′-UTR of the *P. berghei dihydrofolate reductase/thymidilate synthase* (*dhfr/ts*) locus on chromosome 7 was used to detect integration into the correct chromosome.

### Western Blotting

Mixed asexual blood stages were isolated from infected blood after removal of white blood cells using a CF11 (Whatman) column. Blood was lysed in red blood cell lysis buffer (0.15 M NH_4_Cl, 0.01 M KHCO_3_, 1 mM EDTA, pH 7.4). For ookinete preparation, parasites from day 5 post infection mice were placed in 1 ml ookinete medium for 24 hr at 20°C for ookinete production. The parasites were then lysed in red blood cell (RBC) buffer for 30 min and purified on a 63% NycoDenz gradient (v/v in CLB).

Cell lysates for western blot analyses were prepared by re-suspending parasite pellets in a 1∶1 ratio of PBS containing Protease inhibitor (Roche) and Laemmli sample buffer. Samples were boiled for 5 min and separated on a 4–15% SDS-polyacrylamide gel. Proteins were transferred to nitrocellulose membranes (Amersham Biosciences) and immunoblotting performed using the Western Breeze Chemiluminescent Anti-Rabbit kit (Invitrogen) according to the manufacturer’s instructions. As primary antibody a commercially available anti-human type-I-PKG antibody (PK1018, Calbiochem) was used at 1∶5000 [22], which recognizes both PbPKG and PfPKG due to a C-terminal epitope of PKG conserved between the three species. As a loading control a rabbit polyclonal anti-GFP antibody (Invitrogen) was used. Quantification of the band intensities was done using the ImageJ software.

### Phenotypic Analyses and Parasite Maintenance

To initiate infections, blood containing 5×10^6^ parasites was injected i.p. into mice. Parasitemia and gametocytemia were determined on Giemsa stained blood films.

Once asexual parasitemia had reached ∼7% and numbers of gametocytes were comparable 30–50 *Anopheles stephensi* SD 500 mosquitoes were allowed to feed for 20 min on anaesthetised infected mice.

Approximately 20 guts were dissected from mosquitoes on day 14 post feeding and mounted under Vaseline-rimmed cover slips after staining with Hoechst 33342 for 10–15 min to assess mid-gut infection. Oocysts were counted on an AxioCam ICc1 digital camera fitted to a Zeiss AxioImager M2 microscope using a 63x oil immersion objective.

On day 21 post-feeding another 20 mosquitoes were dissected and their guts and salivary glands crushed separately in a loosely fitting homogenizer to release sporozoites, which were then quantified using a haemocytometer.

Mosquito bite back experiments were performed on day 21 post feeding using naïve mice and blood smears were examined for the next 14 days to determine infection.

### Exflagellation Assay

10 µl of parasite infected blood containing mature gametocytes at comparable levels were placed in 50 µl ookinete medium (RPMI1640 with 25 mM HEPES, 20% fetal bovine serum, 10 mM sodium bicarbonate, 50 µM xanthurenic acid at pH 7.6) and incubated at 20°C. For drug testing experiments, increasing concentrations of compound 1, 4-[2-(4-fluorphenyl)-5-(1-methylpiperidine-4-yl)-1*H* pyrrol-3-yl]pyridine, (0, 1.25, 2.5, 5 and 10 µM) or compound 2, 4-[7-[(dimethylamino)methyl]-2-(4-fluorphenyl)imidazo[1,2-*a*]pyridin-3-yl]pyrimidin-2-amine, (0, 0.25, 0.5, 1 and 2 µM) were added to the medium. The number of exflagellation centers per field was counted after 15 min incubation using phase contrast microscopy in 12–15 fields of view using a 63x objective and 10x ocular lens [20].

### Ookinete Conversion Assay

Parasite infected blood containing comparable levels of mature gametocytes was re-suspended in ookinete medium as previously described [20]. For drug testing experiments, increasing concentrations of compound 1; 0, 1.25, 2.5, 5 and 10 µM) or compound 2; 0, 0.25, 0.5, 1 and 2 µM) [8], [9], [12], [22], [23]) were added to the medium prior to addition of blood. After 24 h, cells were re-suspended in fresh ookinete medium containing an anti-P28 Cy3 conjugated 13.1 monoclonal antibody staining the surface of activated female gametocytes and ookinetes and Hoechst 33342 DNA dye before being examined with a Zeiss AxioImager M2 microscope. Ookinete conversion was calculated as the percentage of ookinetes relative to all 13.1 positive cells [28].

### Genetic Complementation Assay

Genetic complementation crosses were carried out between different mutant parasite lines as described previously [20], [21]. Briefly, for complementation of ookinete conversion, mature gametocyte-containing blood from mice infected with different parasite lines was mixed and re-suspended in ookinete medium, and ookinete conversion was determined after 24 h as described above. For complementation of oocyst production, mice were infected with combinations of different parasite strains and 3–6 days old female *A. stephensi* mosquitoes were infected by directly feeding on these mice. Mosquitoes were dissected 12–14 days post infection and the presence of oocysts was determined.

### *In vivo* Assay using Peters Four Day Test

Female mice aged 6–8 weeks (18–20 g) in groups of 5 were used for all experiments. For each construct tested, 3 groups of 5 mice were required (5 for Compound 2, 5 for chloroquine and 5 untreated controls). Animals were randomized and inoculated intravenously with 2×10^6^ red blood cells parasitized with the respective *P. berghei* parasite construct. Animals in the Compound 2 group were dosed twice daily for 4 consecutive days beginning on the day of infection (i.e. D0 to D+3) at 25 mg/kg and animals in the chloroquine group were dosed once daily at 10 mg/kg for 4 consecutive days. The parasitaemia was determined on the day following the final treatment (D+4) and the suppression of parasitaemia was calculated when compared to untreated controls.
